# Novel Cephalosporins in Septic Subjects and Severe Infections: Present Findings and Future Perspective

**DOI:** 10.3389/fmed.2021.617378

**Published:** 2021-05-07

**Authors:** Silvia Corcione, Tommaso Lupia, Francesco Giuseppe De Rosa

**Affiliations:** ^1^Department of Medical Sciences, Infectious Diseases, University of Turin, Turin, Italy; ^2^Tufts University School of Medicine, Boston, MA, United States

**Keywords:** cephalosporin, sepsis, severe infections, blood-stream infections, multi-drug resistant bacteria

## Abstract

In past decade, cephalosporins have developed significantly, and data regarding novel cephalosporins (i.e., ceftobiprole, ceftaroline, ceftolozane/tazobactam, ceftazidime/avibactam, and cefiderocol) within septic and bacteremic subjects are rising. These compounds generally offer very promising *in vitro* microbiological susceptibility, although the variability among gram-negative and -positive strains of different cohorts is noticed in the literature. We require further pharmacological data to measure the best dose in order to prevent sub-therapeutic drug levels in critically ill patients. These new compounds in theory are the sparing solution in the *Enterobacteriales* infection group for different antimicrobial classes such as aminoglycosides notably within endovascular and GNB-bacteremias, as well as colistin and carbapenem-sparing strategies, favoring good safety profile molecules. Moreover, new cephalosporins are the basis for the actual indications to open up new and exciting prospects for serious infections in the future. In future, patients will be addressed with the desirable approach to sepsis and serious infections in terms of their clinical situation, inherent features of the host, the sensitivity profile, and local epidemiology, for which evidence of the use of new cephalosporin in the treatment of severe infections will fill the remaining gaps.

## Introduction

Sepsis has been defined as “one of the oldest and most elusive syndromes in medicine” (1). In 2017 Rudd et al. estimated, worldwide, 48.9 million cases of sepsis and 11.0 million deaths due to sepsis (2). Sepsis complexity resides in a dysregulated host response to an infection and in jeopardy to develop acute organ dysfunction with a high risk of in-hospital mortality rates estimated between 25 and 30% (3). This syndrome requires urgent treatment; thus, an awareness of the presenting characteristics of sepsis is highly important (4). In medicine, we must always look to the past with an eye to the future. From this perspective, this paper evaluates and discusses the available data on novel cephalosporins (i.e., ceftobiprole, ceftaroline, ceftolozane/tazobactam [C/T], ceftazidime/avibactam [C/A], and cefiderocol) (5) in the antimicrobial management of sepsis and severe infections to assess whether these new molecules can provide innovative answers to ancient questions (6).

## Cephalosporins with a Main Anti-MRSA Activity

### Ceftobiprole

Ceftobiprole medocaril is currently approved in Europe as an extended-spectrum cephalosporin for adult community acquired and nosocomial, non-ventilator-associated pneumonia (i.e., CAP and HAP) (7, 8), and skin and soft tissue infection (SSTIs), including diabetic foot infections (9, 10).

Clinical application and actual experiences with ceftobiprole are limited but nevertheless promising for sepsis and bloodstream infections (BSIs) (11, 12). A discreet proportion of gram-positive sepsis was included in phase III trials for CAP, HAP and SSTIs (7–10). Rello et al. (13) developed two interesting grouped analyses—a test of cure (TOC) and mortality—for ceftobiprole, vs. comparators (e.g., vancomycin, linezolid, and ceftazidime) for *Staphylococcus* spp. BSIs through the extrapolation of the clinical data directly from phase III studies (7–10). In the TOC analysis, the clinical cure rate of the ceftobiprole group (48.9 percent, 22/45 patients) was similar to that of the comparators (44.0 percent, 22/50), specifically the subgroups of coagulase-negative staphylococci (CoNS; 45.5 vs. 45.5%), methicillin-sensitive *Staphylococcus aureus* (MSSA; 44.4 vs. 46.7%), and methicillin-resistant *S. aureus* (MRSA; 55.6 vs. 22.2%) (12, 13). Furthermore, the 30-day all-cause mortality in the ceftobiprole group was 8.9% (4/45) vs. 16.0% (8/50) in the comparator group (12, 13). In the ceftobiprole group, death rates were zero for MRSA bacteraemic patients compared with 22.2 per cent in the comparator cohorts (12, 13). Despite the interesting results, the conclusions drawn by Rello et al. had been obtained from a sample of just 18 patients, without a complete analysis of all data in the entire subgroup (13).

Ceftobiprole (alone or in combination) could therefore play an important role in treating endovascular infections due to its high bactericidal activity, favorable resistance profile, and potential synergism with other antigram-positive molecules (11–13). In a rat model of endocarditis, a subtherapeutic dose of ceftobiprole plus vancomycin was as effective against MRSA and vancomycin intermediate-resistant *S. aureus* strains as ceftobiprole standard dose monotherapy (14, 15). Theoretically, ceftobiprole used to treat endocarditis could permit a nephrotoxic-sparing strategy, thus avoiding the aminoglycoside and glycopeptide side effects and the need for therapeutic drug monitoring. Ceftobiprole also plays a vital role in penicillin-allergic subjects with severe gram-positive infections (11–13). A daptomycin-based scheme, complemented with an adjunct of ceftobiprole, also seems promising in clinical applications within endocarditis therapeutic management (16, 17). An ongoing non-inferiority trial (double-blinded and randomized, https://clinicaltrials.gov/ct2/show/NCT03138733) comparing standard dose ceftobiprole and daptomycin (6 mg/kg/24 h) in adult patients with *S. aureus* bacteraemia (including right-sided infective endocarditis) will provide more information (18). One potential concern surrounding ceftobiprole is determining the adequate dosage for bacteraemia and endocarditis. The prospect of accomplishing this target for MRSA strains with the ongoing approved dose is >90% with MIC of 4 mg/L (19–21).

A higher exposure (100% T > MIC) is however associated with strong bactericidal action and therefore is the preferred goal for severe and high inoculum infections (19–21). With the current dose the probability of receiving 100% > T > MIC for MRSA strains will be lower, but this can be improved significantly through the prolonged infusion (over 4 h) or ongoing infusion of patients at higher doses (500 mg/6 h or 1 g/8–12 h) (19–21).

Little is known about CSF penetration for ceftobiprole, which may still be a valuable option in primary meningitis caused by *Streptococcus pneumoniae* (including penicillin- and ceftriaxone-resistant strains) and secondary, post-surgical meningitis requiring both gram-positive and susceptible gram-negative coverage (11–13, 22). The antibacterial activity was comparable to cefepime with β-lactamase-negative strains of *Escherichia coli, Klebsiella pneumoniae*, and *Haemofilus influenzae* in the animal model proposed by Stucki et al. (22). Moreover, the same authors (22) discovered that ceftobiprole reached about 16% of serum levels through inflamed meninges compared to about 2% of serum levels through uninflamed meninges in a rabbit meningitis model, similarly to cefepime pharmacokinetic in CSF.

This fifth-generation cephalosporin has been approved for official indications of CAP and HAP adults in 12 European countries, Canada, and Switzerland (11). Moreover, ceftobiprole blends excellent spectrum with beta-lactam safety for low to moderate MDR HAP pathogenes in frail patients who could be at great risk from adverse effects caused by MRSA or anti-MRSA coverage, including oxazolidinones or glycopeptides (6, 11, 12).

There are promising activities of this novel cephalosporins on MRSA isolates, including Panton-Valentine Leukocidine positive strains, whether or not it is a slight change depending on the type of SCCmec, as shown in recent findings concerning ceftobiprole isolates in Phase III SSTI and pneumonia studies (23, 24). In severe adult CAP, coinfection, or superinfection over viral pneumonia, ceftobiprole can be a suitable option when the risk for MRSA or susceptible *P.aeruginosa* coinfection is high (6, 11, 12).

VAP still represents an area of uncertainty according to clinical trial results (8) due to the inadequate sample size and substantial baseline sample heterogeneous features (6). Ceftobiprole data for BSIs in real life are limited, but promising, despite the need for further pharmacokinetic data in special populations such as patients with critical illness or elevated creatinine clearance (25).

### Ceftaroline

Ceftaroline is a fifth-generation cephalosporin with a peculiar affinity to the penicillin binding protein (PBPs) 2a, an MRSA-specific protein, with an excellent spectrum of activity on common bacterial causes of CAP (26, 27) and SSTIs (28). Ceftaroline has activity against a wide spectrum of gram-positive bacteria including MSSA and MRSA, also including some resistant *S. aureus* strains (vancomycin intermediate, heterogeneous vancomycin intermediate, vancomycin-resistant, or daptomycin non-susceptible) and MDR *S. pneumoniae* (29–31). Moreover, ceftaroline also exhibits activity against a broad group of gram-negative, ESBL-negative, or AmpC-producing *Enterobacteriaceae* (29–31). The US Food and Drug Administration (FDA) approved a label expansion for the treatment of *S. aureus* bacteraemia associated with SSTIs in adults in 2015 and in the pediatric population in 2016 (32). The literature regarding the bacteraemic cohorts of patients treated with ceftaroline (alone or in combination) is growing and has been mostly comprised of subjects with a diagnosis of either persistent bacteraemia or MRSA bacteraemia that is not susceptible to vancomycin or daptomycin (32–47). Persistent bacteraemia (with or without a known focus of infections) is not an uncommon finding, which may be associated with increased mortality and morbidity, and refers to blood cultures that were positive within the same infectious episode and on different days (33, 34). Interesting observations on time to eradication of MRSA in BSIs were extrapolated from a retrospective matched case-control study by Paladino et al. (33): the ceftaroline cohort reported a median time to eradication that was about half the median time of the control group treated with vancomycin (4 vs. 8 days; interquartile range [IQR]: 3.0–7.5 days vs. 5.8–19.5 days; *P* = 0.02). The time to eradication was further reduced to 2 days (IQR: 1–4 days) in Arshad et al.'s (34) multicenter observational study of 211 patients (33). Clinical success in the most representative studies has ranged from 60 to 88% (34–39). Interestingly, Arshad et al. (34) reported a clinical cure rate of 69.7% when ceftaroline was used as a monotherapy and 64.9% when it was used in combination. Furthermore, even a microbiological cure reported good results, between 70 and 100%, but these were variable depending on the time of consideration (34). Mootz et al.'s (45) retrospective comparative effectiveness study included adults hospitalized with sepsis who received ceftaroline or daptomycin within 14 days of hospital admission. Patients treated with ceftaroline were less likely to experience readmission at 30 days (25 vs. 37%, *P* = 0.06), 60 days (27 vs. 44%, *P* = 0.008) and 90 days (28 vs. 46%, *P* = 0.01) compared to those treated with daptomycin (45). Moreover, the ceftaroline group showed a lower in-hospital mortality rate (7 vs. 12%, *P* = 0.4) at 30 days (3 vs. 9%, *P* = 0.1), 60 days (6 vs. 12%, *P* = 0.2), and 90 days (7 vs. 15%, *P* = 0.1) vs. the comparator (45).

Ceftaroline, a rapid BSI clearance beta-lactam, also serves as an interesting solution to complicated endocarditis (46). This is illustrated by the retrospective CAPTURE analysis involving 55 patients receiving ceftaroline treatment with gram-positive endocarditis (47). The overall success rate in this study was 70.9% (47). In particular, ceftaroline therapies were extremely effective as a first-line therapy (75.0%) as well as in patients suffering from right-sided endocarditis (80.8%) and MRSA (77.3%) (47).

Ceftaroline is currently used in the management of persistent gram-positive bacteraemia (36) and, also due to the wide spectrum of microbiological activity comprising gram-negative organisms, may be a feasible choice for catheter-related and -associated BSIs (12, 32). The bactericidal and time-dependent activity of ceftaroline, as well as ceftobiprole, could potentially be implemented in therapeutic schemes for bacteraemia and endovascular infection to improve the safety of kidney function, limiting glycopeptides and the need for therapeutic monitoring, with consistent advantages in centers with scarce laboratory resources (11, 12, 32, 33). Ceftaroline could also be used in the treatment of severe infections, including both primary (e.g., post-traumatic) and secondary (e.g., post-surgical) bacterial meningitis, although few data are available for CSF drug concentrations (48, 49).

Stucki et al. (48) estimated that 15% of the serum level of CSF penetrated inflammatory meninges and about 3% of non-inflamed meninges. Mermer et al. (49) found that in an experimental meningitis model both ceftaroline and vancomycin have similar antibacterial efficacy in treating MRSA. Pani et al. (32) confirmed the use of ceftaroline as the fifth off-label indication for meningitis in their latest systematic research.

## Cephalosporins with Main Anti-Gram-Negative Activity

### Ceftolozane/Tazobactam

C/T is a combination of the renowned β-lactamase inhibitor tazobactam and an innovative anti-pseudomonal cephalosporin (5, 50). The FDA and the EMA have approved C/T for complicated urinary tract infection (cUTI) (51) and complicated intra-abdominal infection (cIAI) (52) at a dose of 1.5 g (ratio of 1.0 ceftolozane to 0.5 tazobactam) every 8 h, with double dosage (3 g; ratio of 2.0 ceftolozane to 1.0 tazobactam) in the phase III study ASPECT-NP for the treatment of NP (53). C/T is active in several multi-drug resistant (MDR) and extensively drug-resistant (XDR) *Enterobacteriales* strains, including ESBL-producting strains and *P. aeruginosa*. (54).

Regarding ESBL strains, C/T has differences depending on the pathogen concerned. C/T, in a study published by Tato et al. (55), seems to have higher activity against ESBL-producing *E. coli* than *Klebsiella* spp. Moreover, among ESBL pathogens, Castanheira et al. (56) have found that C/T retains a lower activity in *bla*_SHV_ isolates (61.1%) than *bla*_CTX−M_ strains (91.2%). All together, these data show that C/T is a major component of a carbapenem-sparing strategy, at least in the empiric setting, even if more evidence is needed to confirm the exact role as a targeted treatment of ESBL infections (57).

An increased risk of hospital mortality has been linked to inadequate initial antibiotic treatment for Pseudomonas aeruginosa BSI (58). From a microbiological perspective, 88 percent (*n* = 615) of isolates were susceptible to C/T in a major multi-center study carried out between 2012 and 2015 for meropenem-non-susceptible *P. aeruginosa* isolates in 32 US medically-based centers (59).

For contrast, additional single-center surveillance studies have shown a 60–94 percent C/T susceptibility of different *P. aeruginosa* isolate populations (60–62).

From a clinical point of view, Bassetti et al. (63) described one of the largest clinical trials using C/T in a multicenter cohort of 101 patients with documented *P. aeruginosa* infection. Sepsis and septic shock were present at diagnosis in 26.7 and 11.9% of patients, respectively, and concomitant *P. aeruginosa* bacteraemia was ruled out in only 15.8% of subjects (63).

The only independent predictor of clinical failure was sepsis (odds ratio [OR] = 3.02, 95% confidence interval [CI]: 1.01–9.2; *P* = 0.05) for patients with clinical success in comparisons to those suffering a clinical failure with multivariate analyses (odds ratio [OR] = 3.02, 95 per cent CI: [CI]: 1.01–9.2; *P* = 0.05) (63).

All together, these data show that C/T may be a valuable option to prevent nephrotoxicity with colistin- or aminoglycoside-sparing regimens including the risk of subtherapeutic dosages associated with reduced renal clearances (64, 65).

CEFTABUSE register (65) results showed a non-significant trend toward more favorable 14-day clinical cure rates in C/T patients than aminoglycoside or colistin (81.3 vs. 56.3%; *P* = 0.11%). A similar pattern was found for crude deaths of 30 days (18.8 vs. 28.1%; *P* = 0.73) and acute kidney injury prevalence (0.0 vs. 25.0%; and *P* = 0.04), favors C/T vs. colistin or aminoglycoside.

Similarly, a retrospective multicenter observational cohort study [644] also found that C/T administration was independently associated with clinical cure (adjusted OR: 2.63; 95% CI: 1.31–5.30) and protected against AKI (adjusted OR: 0.08; 95% CI: 0.03–0.22) without any difference in in-hospital mortality. In addition, a systematic Maraolo et al. (62) study concluded that the therapy C/T could be useful even outside of an accepted setting of indication for difficult-to-treat *P. aeruginosa* infections: BSI is the third commonly indicated off-label (23/130; 17.7%).

### Ceftazidime/Avibactam

C/A is an intravenous combination of a third-generation cephalosporin with the non-β-lactam/β-lactamase inhibitor avibactam (66), with activity against ESBL-producing bacteria, *P. aeruginosa* and KPC or OXA-48 carbapenemase producing bacteria. C/A has been approved in cIAI (67), cUTI (68), and NP (69) therapy as well as infection by microorganisms resistant to ceftazidime, according to a specific trial (RECLAIM, RECAPTURE, REPROVE, and REPRISE study, respectively) and, given the comparator mainly based on carbapenem drug administration, it is also a major component of a carbapenem-sparing strategy. Moreover, real-life data did show clinical efficacy in patients with KPC and OXA-48 carbapenemases. In a prospective study, Sousa et al. (70) described a cohort of 55 patients, 54% of whom presented with severe septic shock or sepsis; moreover, 54 of the 57 isolates were OXA-48-producing *K. pneumoniae*, 46% (26/55) of patients had confirmed bacteraemia, and C/A was mainly given as monotherapy (81%) with a mean duration of 13 days; The 14-day mortality rate was 14% (70). In systematic reviews and meta-analysis with infections by carbapenem-resistant bacteria, Fiore et al. (71) did not observe any difference in the mortality rates between C/A monotherapy or combination therapy (*N* = 503 patients; direct evidence OR: 0.96; 95% CI: 0.65–1.41), with similar findings in the unregistered systematic review by Onorato et al. (72).

A retrospective longitudinal study of 138 patients with KPC-Kp bacteraemia, in which a significantly lower mortality was observed in any patient with C/A than other drugs (36.5 vs. 55.8%, *P* = 0.05), and in which this was the only factor that was substantially correlated with survival, was addressed by Tumbarello et al. (73). In this complex analysis, the authors identified septic shock, neutropenia, Charlson Comorbidity Index ≥3, and recent mechanical ventilation as independent predictors of mortality, whereas C/A was the sole independent predictor of survival (73). In addition, Shields confirmed higher rates of clinical success (*P* = 0.006) and survival (*P* = 0.01) for C/A compared to other regimens and observed higher renal safety compared to aminoglycoside and colistin-containing regimens (*P* = 0.002) (74).

All together, data from official trials and real-life experiences showed that C/A is representing a major component in carbapenem-sparing strategies, including patients infected by KPC or OXA-48 carbapenemases producing bacteria in addition to *P. aeruginosa* and ESBL-producing bacteria (75).

### Cefiderocol

Cefiderocol is an innovative siderophore cephalosporin that was produced to target carbapenem-resistant pathogens, including fermenting and non-fermenting (i.e., *P. aeruginosa* and *Acinetobacter baumannii*) GNB (76, 77). Among such pathogens is also included *Stenotrophomonas maltophilia*, which is intrinsically resistant to carbapenems (76, 78). In the CREDIBLE CR, cefiderocol was studied in a randomized, open-label, prospective, phase III clinical study in individuals with carbapenem-resistant gram-negative bacteria infections, regardless of species or infection site source and including sepsis and BSIs (ClinicalTrials.gov registration: NCT02714595) (79, 80). The clinical cure between cefiderocol and the comparator, defined as best available therapy (BAT), was similar for NP (50 vs. 53%) and BSI (43 vs. 43%). Besides, in complicated UTIs, cefiderocol was not inferior to the BAT group in microbiological eradication (53 vs. 20%) (80). In the cefiderocol group, more numerical deaths occurred, especially in the *Acinetobacter* spp subset, a finding which was not unequivocally explained. These results endorse cefiderocol as an alternative for treating patients with limited treatment options of carbapenem-resistant infections (80).

Wunderink et al. (81) recently published an APEKS-NP randomized double-blind, phase III, non-inferiority analysis in which 148 subjects and 152 subjects were allocated, respectively, for cefiderocol and meropenem. The authors suggested that cefiderocol was non-inferior in patients with Gram-negative NP equal to high dose extended-infusion meropenem and similar for all cases-mortality on Day 14 (12.4 vs. 11.6%) (81). The findings indicate that cefiderocol is a promising solution for treating NP, including those caused by MDR gram-negative organisms (81).

Hsueh et al. (82) measured *in vitro* cefiderocol, C/T, and C/A microbiological profile for *P. aeruginosa* and *S. maltophilia* and *A.baumannii* isolates in the bloodstream. In comparison with the C/T and C/A, Cefiderocol demonstrated much greater *in vitro* activity with MICs ≤ 4 mg/L for *P. aeruginosa* isolates resistant to colistin or imipenem (82).

Presently, in order to improve therapeutic effectiveness in serious CRE infections, high levels and combining methods which may have a new inhibitor β-lactam/β-lactamase are likely to be considered (77, 78, 83). Cefiderocol has been added to these therapeutic options, increasing the antimicrobial spectrum to *A. baumannii* and *S.maltophilia*, which in the last decade have been frequently omitted from the new molecules, theoretically allowing for enhanced individualization based on molecular resistance phenotypes, disease severity, susceptibility profiles, and patient characteristics in antimicrobial strategies (77, 78, 83, 84).

Besides, cefiderocol, from a pharmacokinetic point of view, is unique for its dosing regimen to include patients with an augmented renal clearance which is the primary cause for underdosing in very sick patients for beta-lactams.

## Discussion

The creation of three fifth-generation cephalosporins has made a major evolution in the last decade (i.e., ceftobiprole, ceftaroline, and ceftolozane), along with the development of cefiderocol, with its new “trojan horse” active transport mechanism for entering multi-drug resistant bacteria, as well as through novel therapeutic binomials (C/T and C/A) (5, 11, 44, 79). Data on novel cephalosporins may also be available, in addition to the official registration trials, in patients with bloodstream infections and severe infections as well as in infections by MDR bacteria including carbapenemases-producing bacteria from real-life settings. In addition, the future of antimicrobial stewardship in septic and bacteremic patients can be assessed through a multilevel evaluation from the microbiological, pharmacological, clinical, and financial perspectives (5, 12, 77, 78). Although the literature is clear on specific variability in gram-negative and gram-positive strains from various countries and cohorts, as well as different rates of carbapenem resistance, the *in vitro* susceptibilities of the novel cephalosporins are generally very promising. (5, 12, 77, 78). This variability makes it crucial to know their global and local epidemiologies, particularly in MDR gram-negatives with limited treatment options. Furthermore, there is a critical need for more pharmacological data to assess the best dosage and administration modalities in critically ill patients in order to avoid subtherapeutic levels of the drug (5, 12, 77, 78).

**Figure 1:**
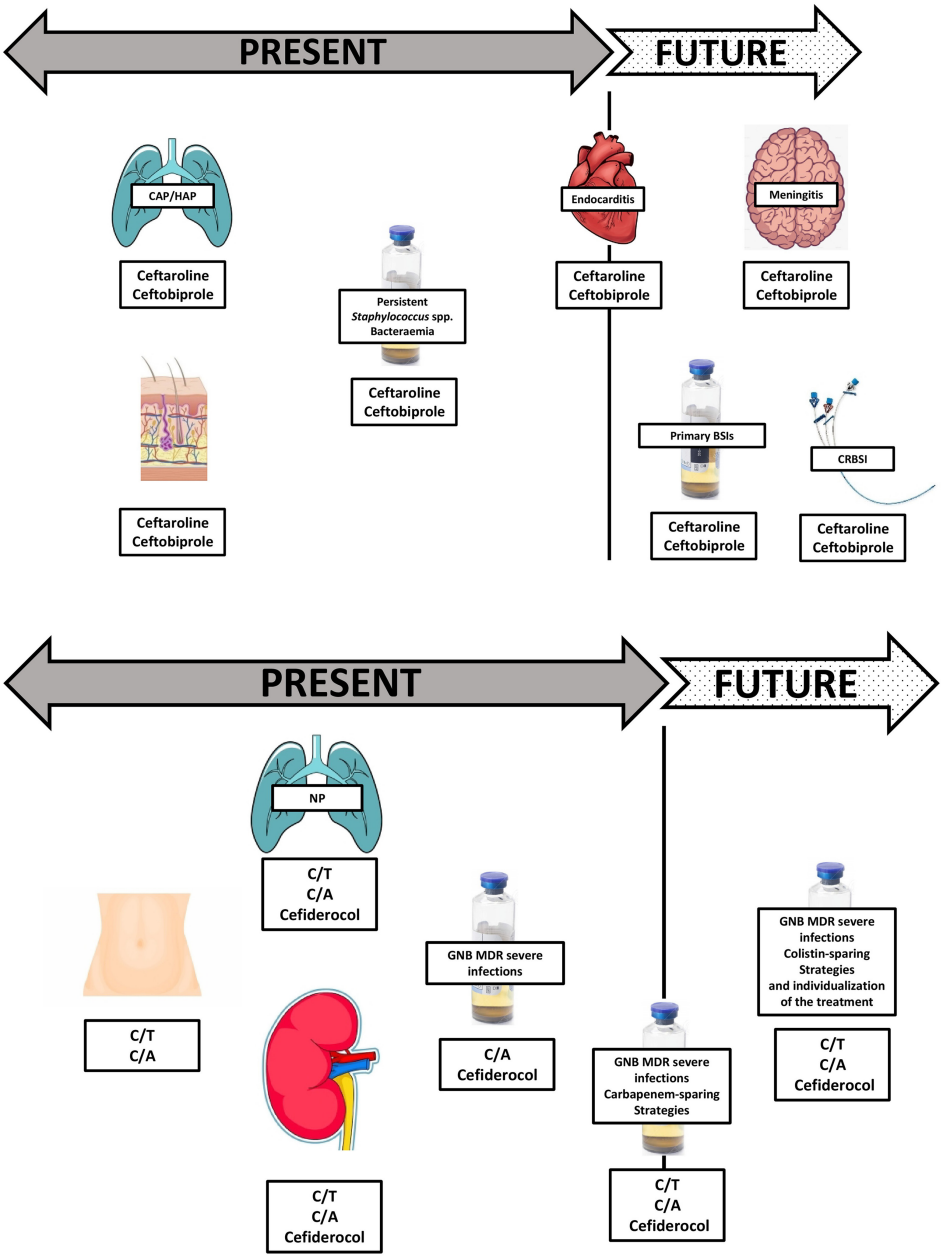
Present and future perspectives within novel cephalosporins compounds.

Theoretically, such new compounds allow a sparing approach in different antimicrobial classes, such as carbapenems, aminoglycosides, colistin, and also vancomycin for ceftaroline and ceftobiprole (12, 65, 77, 78). We are presented with molecules with an increasingly strong pathogen-specific identity, such as C/T for MDR/XDR *P. aeruginosa* and cefiderocol for difficult-to-treat CR strains, and we face a great change in the management of MRSA and endovascular bacteremia due to the evolving clinical data on ceftaroline and ceftobiprole (5, 11, 44, 79). Time seems to be on the side of these novel cephalosporins, making them useful in several severe infections (Figure 1). These molecules can also be used in therapeutic combinations, under particular circumstances. The primary rationale for using two distinct classes of antibiotics with activity against a single pathogen is, on the one hand, to potentiate pathogen clearance and, on the other, to assure the pathogen's susceptibility to the empiric therapy (85, 86). Conversely, monotherapy reduces the risk of antibiotic pressure, the rate of new infections, antibiotic antagonism, toxicity, and costs, though it may not cover MDR pathogens (Table 1) (85, 86). Combination antibiotic treatment was recommended in international guidelines for primary management of septic shock to provide appropriate empirical antibiotic coverage in a scenario with high risk of MDR pathogens (87). However, several other studies found no superiority of combination treatment, and some analyses showed an increased rate of side effects in patients receiving combination therapy (88–90). The desired approach to sepsis and serious infections would be presented to patients in the future based on their clinical condition, host characteristics, susceptibility profiles, and local epidemiology, which would fill the gaps in the use of new cephalosporins that currently exist. Finally, literature data emphasize the small spread of every study and also stress the importance of local monitoring. When determining early use of these agents in severely ill patients, careful consideration should be given to local susceptibility patterns.

**Table 1:** Advantages of monotherapy and combination antimicrobial therapy.

**PROs of Monotherapy**	**PROs of Combination Therapy**
Low antibiotic pressure	Avoid resistance development in difficult-to-treat infections
Low risk of toxicities	Active on different mechanism
Improve de-escalation approach	In MDR infections to ensure sensitivity
Improve antibiotic stewardship bundle	Accelerate pathogen clearance high bacterial loads
Reduce the risk of antibiotic antagonism	Improve synergy between molecules
Improve diagnosis	Decrease the risk of inappropriate empiric antibiotic therapy

## Data Availability Statement

The original contributions presented in the study are included in the article/supplementary material, further inquiries can be directed to the corresponding author/s.

## Author Contributions

All authors listed have made a substantial, direct and intellectual contribution to the work, and approved it for publication.

## Conflict of Interest

The authors declare that the research was conducted in the absence of any commercial or financial relationships that could be construed as a potential conflict of interest.
